# Spectrophotometric and HPLC Methods for Simultaneous Estimation of Amlodipine Besilate, Losartan Potassium and Hydrochlorothiazide in Tablets

**DOI:** 10.4103/0250-474X.62239

**Published:** 2010

**Authors:** S. B. Wankhede, K. C. Raka, S. B. Wadkar, S. S. Chitlange

**Affiliations:** Department of Pharmaceutical Chemistry, Padmashree Dr. D. Y. Patil Institute of Pharmaceutical Sciences and Research, Sant Tukaram Nagar, Pimpri, Pune-018, India

**Keywords:** Amlodipine besilate, area under curve method, hydrochlorothiazide, losartan potassium, reverse phase high performance liquid chromatography, simultaneous equation method, area under curve method

## Abstract

Two UV-spectrophotometric and one reverse phase high performance liquid chromatography methods have been developed for the simultaneous estimation of amlodipine besilate, losartan potassium and hydrochlorothiazide in tablet dosage form. The first UV spectrophotometric method was a determination using the simultaneous equation method at 236.5, 254 and 271 nm over the concentration range 5-25, 10-50 and 5-25 μg/ml for amlodipine besilate, losartan potassium and hydrochlorothiazide, respectively. The second UV method was a determination using the area under curve method at 231.5-241.5, 249-259 and 266-276 nm over the concentration range of 5-25, 5-25 and 10-50 μg/ml for amlodipine besilate, hydrochlorothiazide and losartan potassium, respectively. In reverse phase high performance liquid chromatography analysis is carried out using 0.025 M phosphate buffer (pH 3.7):acetonitrile (57:43 v/v) as the mobile phase and Kromasil C18 (4.6 mm i.d×250 mm) column as stationery phase with detection wavelength of 232 nm linearity was obtained in the concentration range of 2-14, 20-140 and 5-40 μg/ml for amlodipine besilate, losartan potassium and hydrochlorothiazide, respectively. Both UV-spectrophotometric and reverse phase high performance liquid chromatography methods were statistically validated and can be used for analysis of combined dose tablet formulation containing amlodipine besilate, losartan potassium and hydrochlorothiazide.

Amlodipine besilate (AMLO), chemically is [3-ethyl-5-methyl(4RS)-2-[(2-aminoethoxy)methyl]-4-(2-chlorophenyl)-methyl-1-dihydropyridine-3,5-dicarboxylate benzenesulfonate[1]. It is a long acting calcium channel blocker used as an antihypertensive agent. Losartan potassium (LOS), chemically, is 2-butyl-4-chloro-1-[p-(o-1H-tetrazol-5-ylphenyl)benzyl]imidazole-5-methanol monopotassium[2] salt. It is an angiotensin II receptor blocker and chemically is used as an antihypertensive agent. Hydrochlorothiazide (HCTZ), 6-chloro-3,4-dihydro-2H-1,2,4-benzothiadiazine-7-sulfonamide[3] is used as a diuretic. AMLO is official in BP, LOS is official in IP and USP, whereas HCTZ is official in BP and IP. These three drugs are marketed as combined dose tablet formulation in the ratio of 05:12.5:50 mg (AMLO:HCTZ:LOS). Literature survey revealed that a number of methods have been reported for estimation of AMLO, LOS and HCTZ individually or in combination with other drugs[4–18]. However, there is no analytical method reported for the simultaneous estimation of these drugs in a combined dosage formulation. Present work describes rapid, accurate, reproducible, and economical methods for simultaneous estimation of these drugs in tablet formulation. For UV-spectrophotometric method double-beam Shimadzu UV/Vis spectrophotometer, 1700 Pharmaspec, with spectral bandwidth of 2 nm, wavelength accuracy of ±0.5 nm and a pair of 1-cm matched quartz cells, was used. For high performance liquid chromatographic method Merck Hitachi with L-7100 double reciprocating pump, L-7400 UV detector with Winchrom software for data processing was used. Standard gift sample of AMLO was received from Emcure Pharmaceuticals Ltd, Pune, India, LOS as gift sample as from Cipla Ltd, Patalganga, India and HCTZ from Unichem Laboratories, Baddi, India. Combined dose tablet formulation Trilopace, of Sun Pharmaceutical Industries, Dadra, India, containing AMLO (5 mg), LOS (12.5 mg) and HCTZ (50 mg) was purchased from a local pharmacy Store. Methanol used for UV-spectrophotometric method was of AR grade. Acetonitrile (Universal Lab., Mumbai) and other chemicals (Research Lab., Mumbai) used for preparation of buffer solution in RP-HPLC were of HPLC grade, were procured from the local market.

In the UV-spectrophotometric methods, simultaneous equation method (method A), standard stock solutions of AMLO (100 μg/ml), HCTZ (100 μg/ml) and LOS (100 μg/ml) were prepared in methanol. For the selection of analytical wavelength solutions of AMLO (2 μg/ml), HCTZ (5 μg/ml) and LOS (20 μg/ml) were prepared separately by appropriate dilution of standard stock solution and scanned in the spectrum mode from 200 to 400 nm. From the overlain spectra of these drugs (fig. 1), wavelengths 236.5 nm (λ_max_ of AMLO), 254 nm (λ_max_ of LOS) and 271 nm (λ_max_ of HCTZ) were selected for analysis. The calibration curves for AMLO, HCTZ and LOS were prepared in the concentration range of 5-25 μg/ml, 5-25 μg/ml and 10-50 μg/ml, respectively at the selected wavelengths. Absorptivity values were determined for AMLO, HCTZ and LOS and were found to be 32.20/13.36/3.09, 10.86/19.07/61.14 and 37.24/26.55/12.74 at 236.5/254/271 nm, respectively. Using these absorptivity values following Eqns. were developed for determining concentration of AMLO, HCTZ and LOS in tablet sample solution.

(1) $$A_{1}=32.20C_{\mathrm{AMLO}}+10.86C_{\mathrm{HCTZ}}+37.24C_{\mathrm{LOS}}$$

(2) $$A=13.36C_{\mathrm{AMLO}}+19.07C_{\mathrm{HCTZ}}+26.55C_{\mathrm{LOS}}$$

and

(3) $$A_{3}=3.09C_{\mathrm{AMLO}}+61.14C_{\mathrm{HCTZ}}+12.74C_{\mathrm{LOS}}$$

where A1, A2 and A_3_ are absorbance of the tablet sample solution at 236.5, 254 and 271 nm, respectively. C_AMLO_ is the concentration of AMLO, C_HCTZ_ is the concentration of the HCTZ, and C_LOS_ is the concentration of the LOS. For estimating AMLO, HCTZ and LOS in tablet formulation, twenty tablets were weighed and average weight was calculated. The tablets were crushed to obtain fine powder. Tablet powder equivalent to 75 mg of LOS was transferred to 50.0 ml volumetric flask added 30 ml of methanol, sonicated for 20 min and volume was made up to the mark with methanol. The solution was then filtered through Whatmann filter paper No. 41. The filtrate was appropriately diluted with the same solvent to obtain final concentration within Beer Lambert's range for each drug. Absorbance of diluted sample solution was measured at selected wavelengths. The concentration of drugs was determined by using the Eqns 1, 2 and 3. Results of analysis of tablet formulation mentioned in Table 1.

**Fig. 1:**
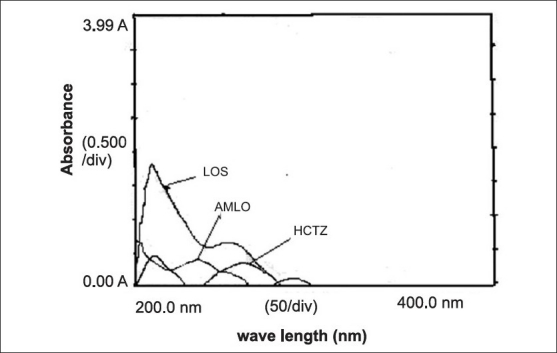
Overlain spectra of AMLO, HCTZ and LOS. AMLO is amlodipine besilate, LOS is losartan potassium and HCTZ is hydrochlorothiazide

**TABLE 1:** ANALYSIS OF TABLET FORMULATION

Method	Component	Label Claim (mg/tab)	Amount Found	% Label Claim*	SD	CV
A	AMLO	05	5.02	100.40	0.8042	0.8012
	HCTZ	12.5	12.51	100.08	0.1824	0.1823
	LOS	50	50.05	100.10	0.1189	0.1188
B	AMLO	05	5.03	100.60	0.8672	0.8620
	HCTZ	12.5	12.52	100.16	0.1237	0.1235
	LOS	50	49.98	99.96	0.0738	0.0738
C	AMLO	05	4.97	100.21	0.8810	0.8791
	HCTZ	12.5	12.498	99.98	1.0073	1.0075
	LOS	50	50.05	99.35	0.9165	0.9225

AMLO is amlodipine besilate, LOS is losartan potassium and HCTZ is hydrochlorothiazide

* Average of six determinations, SD - Standard Deviation, CV-Coefficient of Variation.

In the area under curve method (method B), preparation of standard stock solution was same as mentioned in method A. From the overlain spectra of drugs (fig. 1) wavelengths range 231.5-241.5 nm (AMLO), 266-276 nm (HCTZ) and 249-259 nm (LOS) were selected for the analysis. The calibration curves for AMLO, HCTZ and LOS were prepared in the concentration range as mentioned in method A at the selected wavelength range. Absorptivity values were determined for AMLO, HCTZ and LOS and were found to be 313.28/374.37/30.96, 153.25/200.57/581.14 and 374.37/264.49/123.96 at 231.5-241.5/266-276/249-259 nm, respectively. Using these absorptivity values following equations were developed for determining concentration of AMLO, HCTZ and LOS in tablet sample solution.

(4) $$A_{1}=313.28C_{\mathrm{AMLO}}+153.25C_{\mathrm{HCTZ}}+374.37C_{\mathrm{LOS}}$$

(5) $$A_{2}=374.37C_{\mathrm{AMLO}}+200.57C_{\mathrm{HCTZ}}+264.49C_{\mathrm{LOS}}$$

and

(6) $$A_{3}=30.96C_{\mathrm{AMLO}}+581.14C_{\mathrm{HCTZ}}+123.96C_{\mathrm{LOS}}$$

where A1, A2 and A_3_ are area under curve of the sample at 231.5-241.5, 266-276 and 249-259 nm, respectively, C_AMLO_ is the concentration of AMLO, C_HCTZ_ is the concentration of the HCTZ, and C_LOS_ is the concentration of the LOS. Preparation of sample solution for analysis of tablet formulation was same as described under method A. The concentrations of AMLO, HCTZ and LOS were determined by using the Eqns. 4, 5 and 6. Result of analysis of tablet formulation are mentioned in Table 1.

In the reverse phase high performance liquid chromatography (method C), following optimum conditions were established for quantitative analysis of AMLO, HCTZ and LOS in tablet formulation by trial and error. Mobile phase: a mixture of phosphate buffer (0.025M, pH-3.7 adjusted with ortho phosphoric acid) and acetonitrile in the ratio of (57:43 v/v), column: Kromasil C18 (4.6 mm i.d×250 mm), flow rate: 1.0 ml/min for 6.3 min then 1.3 ml/min from 6.3 min onwards, detection wavelength: 232 nm, temperature: room temperature. Sample solution of AMLO, HCTZ and LOS 6 μg/ml, 15 μg/ml and 60 μg/ml, respectively were prepared in mobile phase and chromatographed under optimum chromatographic conditions. The proposed chromatographic conditions were found suitable for effective separation and quantitation of AMLO (RT-5.12 min), HCTZ (RT-3.42 min) and LOS (8.02 min) with resolution of 2.32 (between HCTZ and AMLO) and 7.91 (between AMLO and LOS), tailing factor- 1.5 for AMLO, 1.33 for HCTZ and 1.05 for LOS. The calibration curves (mean peak area Vs concentration) for AMLO, HCTZ and LOS were prepared in the concentration range of 2-14 μg/ml, 5-40 μg/ml and 20-140 μg/ml, respectively at 232 nm. For the estimation of these drugs in the tablet formulations, twenty tablets were weighed and average weight was calculated. The tablets were crushed to obtain fine powder. Tablet powder equivalent to 50 mg of LOS was transferred to 50.0 ml volumetric flask added 30 ml mobile phase and ultrasonicated for 20 min, volume was then made up to the mark with mobile phase. The solution was then filtered through a Whatmann filter paper No. 41. The filtrate was appropriately diluted with the mobile phase to obtain final concentration within linearity range for each drug in the ratio 60:15:6 μg/ml LOS: HCTZ: AMLO, respectively. The contents were mixed thoroughly and filtered through a 0.2 μ filter. Twenty microlitres of solution was injected and chromatographed under optimum chromatographic condition. A typical chromatogram of AMLO, LOS and HCTZ is shown in (fig. 2). The concentration of AMLO, HCTZ and LOS in tablet sample solution was determined by comparing the peak area of the sample with that of standard at 232 nm. Results of analysis tablet formulation are shown in Table 1.

**Fig. 2:**
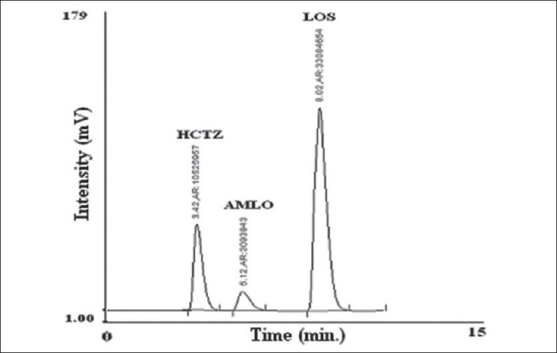
Typical chromatogram of standard solution of AMLO, HCTZ and LOS. AMLO is amlodipine besilate, LOS is losartan potassium and HCTZ is hydrochlorothiazide

Accuracy of the proposed UV-spectrophotometry and reverse phase high performance liquid chromatography method was studied by performing recovery studies by standard addition method at three different levels 80%, 100% and 120%. The results of recovery studies, expressed as % recovery, are mentioned in Table 2. Robustness of the proposed methods was studied by analyzing tablet formulation under varied conditions like different analysts, on different days and different times on same day. The standard deviation for analysis under different conditions was below 2% indicating robustness of method. Ruggedness of the reverse phase high performance liquid chromatography method was studied by deliberately changing the method parameters viz. change in mobile phase composition (±1 ml) and change in flow rate (±0.1 ml/min). Reverse phase high performance liquid chromatography method was found to withstand these variations as there was no significant change in retention time, tailing factor and resolution.

**TABLE 2:** RESULTS OF RECOVERY STUDIES

Methods	Level of % recovery	% Recovery*			Standard Deviation			Relative standard deviation		
			
		AMLO	HCTZ	LOS	AMLO	HCTZ	LOS	AMLO	HCTZ	LOS
A	80	99.78	100.75	99.81	0.5869	0.2758	0.0070	0.5882	0.2737	0.0070
	100	101.16	100.74	99.68	0.9122	0.3536	0.2192	0.9017	0.3510	0.2199
	120	100.05	100.45	99.91	0.2192	0.0212	0.0566	0.2191	0.0211	0.0567
B	80	99.35	100.14	99.97	0.1768	0.2121	0.0778	0.1780	0.2118	0.0778
	100	99.51	100.11	100.05	0.0707	0.0919	0.0212	0.0710	0.0918	0.0212
	120	99.33	99.92	100.05	0.1061	0.0989	0.0070	0.1068	0.0989	0.0070
C	80	100.37	100.51	100.40	0.5515	0.2051	0.4243	0.5495	0.2041	0.4226
	100	100.71	100.58	100.07	0.0212	0.3182	0.1485	0.0211	0.3164	0.1484
	120	99.47	99.85	99.73	0.2263	0.0070	0.2828	0.2275	0.0070	0.2836

AMLO is amlodipine besilate, LOS is losartan potassium and HCTZ is hydrochlorothiazide

* Average of three determinations, SD - Standard Deviation, CV-Coefficient of Variation.

Based on the results obtained, it is found that the proposed UV-Spectrophotometric and reverse phase high performance liquid chromatography methods are accurate, precise, reproducible economical and can be employed for routine analysis of AMLO, LOS and HCTZ in combined dose tablet formulation.
